# **A**utomated measurement of macular neovascularization lesion size in nAMD using AI segmentation

**DOI:** 10.1007/s00417-025-07007-0

**Published:** 2025-11-17

**Authors:** Anna Vahldiek, Lukas Heine, Benja Vahldiek, Jasper Schröter, Jan-Niklas Wolf, Michael Swora, Lars Reissberg, Laurenz Pauleikhoff, Jens Kleesiek, Daniel Pauleikhoff

**Affiliations:** 1 Institute for AI in Medicine, University Medicine Essen, Essen, North-Rhine Westfalia Germany; 2 Institute for AMD Research, Hohenzollernring 60, 48145 Münster, Germany; 3 Department of Ophthalmology, University Hospital Hamburg-Eppendorf, Hamburg, Germany

**Keywords:** Neovascular AMD, Anti-VEGF therapy, Artificial intelligence, OCT

## Abstract

**Purpose:**

To compare artificial intelligence (AI)-based annotations of hyperreflective material (HRM) and manual demarcation of macular neovascularization (MNV) on optical coherence tomography (OCT) volume scans in neovascular age-related macular degeneration (nAMD), and to assess the suitability of AI-driven OCT segmentation for longitudinal lesion monitoring.

**Methods:**

In this retrospective study, 42 eyes from 36 patients (21 f, 15 m; mean age baseline 76.6 y) with exudative nAMD were analyzed using longitudinal spectral-domain OCT data. Manual MNV demarcations on *en-face* OCT projections served as ground truth and were compared to AI-predicted HRM segmentations generated by a 3D nU-Net model on OCT scans. HRM and MNV lesion areas were quantified at multiple time points, and agreement between manual and AI-based measurements was evaluated using Pearson correlation, ordinary least squares regression and robust regression.

**Results:**

A highly similar mean lesion growth was observed when comparing HRM/MNV lesion sizes in longitudinal measurements. Point-by-point comparison revealed a strong overall correlation (r = 0.78) between AI-predicted and manually annotated HRM areas with increasing significance with longer follow-up. However, two aspects were responsible for some AI measurements being larger than manual measurements: At baseline, AI measurements included hyperreflective subretinal fluid as HRM, which was resorbed after three anti-VEGF injections, and during longer-term follow-up, manually annotated MNV areas were occasionally smaller than those derived from AI-based HRM segmentation due to the manual underestimation of very thin HRM.

**Conclusions:**

AI-based segmentation of HRM on OCT scans demonstrates strong overall agreement with manual MNV measurements, particularly on longitudinal assessments. Despite some AI-based overestimations occurring at baseline and some manual MNV underestimations during follow-up, measurements between both methods were highly comparable over time.

## Key messages

***What is known***

- Quantifying macular neovascularization (MNV) lesion size in neovascular age-related macular degeneration (nAMD) is essential for characterizing disease severity.
- Manual delineation of MNV on OCT scans is time-consuming, whereas AI-based segmentation enables automated, precise and reproducible quantification.

***What is new***

- We demonstrate that automated measurements of hyperreflective material (HRM) size correlate well with manual MNV size annotations over time.
- AI-based automated measurements of HRM size can be used as a surrogate for MNV size and enables automated, precise and reproducible quantification of HRM/MNV over time.

## Introduction

Exudative neovascular age-related macular degeneration (nAMD) is a leading cause of irreversible vision loss in aging populations, driven by the pathological formation of macular neovascularization (MNV) and exudation of fluid under the retinal pigment epithelium (RPE) as well as underneath and in the retina [1–5]. Accurate quantification of MNV lesion size and progression is essential for individualized treatment decisions and evaluation of treatment response [6].

In geographic atrophy (GA), traditionally, fundus autofluorescence (FAF) and optical coherence tomography (OCT) have been the primary non-invasive imaging modalities used to assess structural changes in the retina [7, 8]. While FAF provides a planar overview of retinal alterations, OCT offers high-resolution, cross-sectional visualization of retinal architecture and pathology [9]. Ehlers et al. demonstrated a strong correlation between areas of GA measured manually on FAF images and those derived from OCT images, validating the clinical utility of cross-modality comparisons and proposing OCT as the more sensitive and detailed method for atrophic disease monitoring. However, the authors also highlighted the practical limitations of manual OCT-based quantification: the segmentation and measurement of structural biomarkers on volumetric OCT data remain time-consuming and labor-intensive, limiting their scalability in both clinical and research settings [10].

In exudative nAMD, fluorescein angiography (FA) and OCT have also been the primary diagnostic imaging modalities used [11]. MNV appears on FA as hyperfluorescent lesions with progressive leakage, and on OCT as hyperreflective material (HRM), often accompanied by sub-RPE, subretinal, and intraretinal fluid [12]. To described morphologic MNV changes during anti-angiogenic therapy, size measurements of the MNV are of need [6].

Therefore, in previous studies, size progression of MNV using manual demarcation on OCT volume scans, particularly under a reactive treatment regimen such as the IVAN study protocol, was observed [6, 13]. These studies demonstrated that MNV size, especially its change over time during long-term therapy, may serve as a potential biomarker for disease activity and treatment response in individual cases of nAMD. However, manual delineation of MNV across sequential OCT volume scans - combined with *en-face* infrared (IR) imaging - is subjective and also time-consuming and labor-intensive [10, 14, 15]. Thus, the implementation of AI-based methods offers a promising, reliable, and reproducible alternative to manual assessment of MNV size and its progression [16–18] and we previously described a 3D convolutional architectures, particularly nnU-Net, yielded the best results in detecting HRM lesions across volumetric nAMD OCT datasets [19].

The aim of the present study was to investigate whether AI-based quantification of hyperreflective material (HRM) located beneath the retinal pigment epithelium (sub-RPE HRM) or beneath the retina (subretinal HRM) provides a measurement of macular neovascularization (MNV) that is comparable to manual assessment. This comparison is relevant because both manual MNV measurements (based on subjective evaluation of OCT B-scans and en face projections) and AI-based HRM quantification have inherent limitations. However, if AI-based measurements reflect changes in MNV size in a manner similar to manual quantification, such automated MNV characterization could be integrated into clinical routine for the assessment of therapeutic effects.

## Methods

### Study design and data acquisition

The present analysis is based on patients from an observational treatment study performed by AMD-Netz Münster [20]. Treatment naïve eyes with exudative nAMD were included and treated according to German nAMD treatment guidelines (Deutsche Ophthalmologische Gesellschaft (DOG), Retinologische Gesellschaft e. V. (RG), Berufsverband der Augenärzte Deutschlands e.V. (BVA) [21, 22]. For this comparative study, eyes that had received continuous treatment for a duration of three to eight years were selected. The study protocol was retrospective in nature and included anonymized data evaluation, was approved by the local Institutional Ethics committee (file number 2017–033-f-S) and adhered to the tenets of the Declaration of Helsinki for research involving human subjects. The anonymized data was accessed for research purposes starting in 2023.

The nAMD diagnosis of all eyes was confirmed by a retina specialist (D. P.) based on imaging acquired using a Heidelberg Retina Angiograph 2 (HRA2) and a Heidelberg Spectralis OCT (both Heidelberg Engineering, Heidelberg, Germany). Patients were treated according to an “as needed” or “pro re nata” (PRN) protocol as described in the IVAN study [23]. Treatment was performed with either bevacizumab, ranibizumab or aflibercept at the treating physician’s discretion. In case of insufficient response, a switch between agents was possible.

Each OCT volume scan consisted of at least 49 B-scans, covering a 6 × 6 × 2 mm region centered on the fovea. Automatic real-time (ART) averaging with values greater than 12 was employed to enhance image quality, and automatic foveal centering was used to ensure consistent spatial alignment across visits.

### AI model development and validation

The semantic segmentation model used for automated hyperreflective material (HRM) measurement was developed and validated in a prior study, with a detailed description of its architecture, training, and performance published in [19]. For clarity and completeness, we summarize the key aspects here.

Model Architecture and Training: The model is a 3D nnU-Net, an architecture renowned for its strong performance in biomedical image segmentation [24]. The model was trained on a dataset of 20 OCT volume scans from patients with nAMD, which are excluded from subsequent analyses.

Ground Truth Annotation and Inter-Rater Agreement: The ground truth annotations for model training were created by a team of four trained graders from a specialized AMD reading center under the supervision of a senior ophthalmologist (D.P.). The annotation scheme was comprehensive, including anatomical retinal structures and pathologies, with HRM being one of the key labels. To ensure the reliability of these ground truth annotations, a formal inter-rater agreement analysis was conducted. Four annotators independently segmented a representative OCT scan, and the consistency of their labels was measured using the Dice Similarity Coefficient (DSC). The overall mean DSC across all structures was 0.943, indicating substantial agreement. For the specific segmentation of HRM, the mean DSC was 0.884 ± 0.038, confirming that the ground truth data used for training the AI model is consistent and reliable.

Model Performance: The segmentation accuracy of the model was rigorously evaluated on the unseen hold-out test set. The 3D nnU-Net achieved a mean DSC of 0.894 ± 0.007 for the segmentation of HRM. This high level of spatial overlap between the AI-generated masks and the manual ground truth confirms that the model accurately identifies both the location and the shape of HRM lesions, providing a solid foundation for the quantitative analyses performed in this study.

### Manual MNV size measurements on OCT

**Fig. 1:**
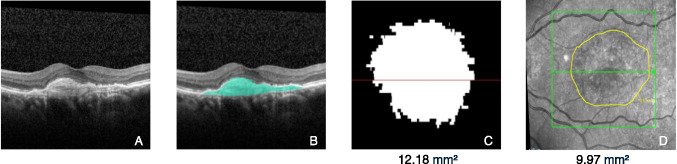
Example of manual and AI-generated assessment of a MNV/HRM. HRM area measured in square millimeter (**A** = foveal OCT-B scan; **B** = foveal OCT B-scan with AI-generated HRM annotation; **C** = AI-generated *en-face* presentation of HRM; **D** = manual *en-face* demarcation of MNV)

To assess the MNV size on OCT manually, MNV lesions were identified by an experienced retina specialist (D.P.) on OCT volume scans (each B-scan was evaluated) based on a correlation of FA features of MNV and OCT features. These included flat and irregular pigment epithelial detachment in case of type 1 MNV or subretinal hyperreflective material not representing hyperreflective fluid in case of type 2 MNV [6, 25]. To measure MNV size, its borders were manually transposed onto the corresponding *en-face* near infrared image (NIR) using the in-built region finder software (Heidelberg Engineering, Heidelberg, Germany) (Fig. 1). The quantification of MNV on OCT is subject to certain limitations, the principal one being the challenge of differentiating hyperreflective MNV from hyperreflective fluid containing fibrin and other components. This ambiguity affects manual measurements and is particularly relevant for AI-based measurements of HRM areas. To address this in the manual workflow, care was taken to exclude discernible hyperreflective fluid from the delineated MNV area at baseline. However, this manual approach is ultimately constrained by the subjectivity inherent in demarcating MNV on B-scans and transposing those boundaries onto the en-face image with the region finder software.

MNV size was measured at treatment initiation, after three months, after one year and after each subsequent year of treatment. For each patient, measurements of MNV size were manually exported into Microsoft Excel (Microsoft Corporation, Redmond, WA, USA).

### AI-based HRM measurements on OCT

To compare the manually measured MNV size with AI-based HRM measurements, a semantic segmentation model was trained on Spectralis-based OCT volume scans with exudative nAMD. The ground truth annotations were created by a team of four trained graders from an AMD reading center under the supervision of D.P.. The labeling process was part of a broader multi-class segmentation effort that included HRM as well as 15 additional retinal and subretinal structures and pathologies. All annotations were performed using the ImFusion labeling software (version 0.24.3) following standardized protocols and supported by visual reference examples to ensure consistency across cases. HRM was defined on OCT scans as well-demarcated hyperreflective material located within the outer retina or subretinal space, consistent with MNV-associated pathology [5, 12, 26]. A critical caveat is that the AI-generated HRM segmentations – as mentioned before—may include both hyperreflective MNV and confounding hyperreflective fluid. This is particularly relevant at baseline, as differentiating between these two features on pre-treatment OCT B-scans is challenging. Following anti-VEGF therapy, the resorption of fluid allows the remaining HRM to serve as a more specific correlate for the MNV lesion. Consequently, baseline HRM measurements should be interpreted with caution, as they may overestimate the true MNV area due to this fluid component.

To ensure high-quality and consistent labeling, all segmentations were reviewed by experienced readers. Inter-observer agreement was monitored during the annotation process. Although these manual annotations are referred to as "ground truth," it is acknowledged that they represent an approximation rather than an absolute standard, due to inherent variability in human interpretation. To address this limitation, the annotation process included frequent cross-checks and iterative feedback rounds. Additionally, different two-dimensional (2D) and three-dimensional (3D) AI network structures were tested with these annotations in respect to different morphologic parameters and the best performing network, a 3D nnUNet [24], was used for the present AI-based HRM measurements (Fig. 1). For details, please refer to [19].

### Analyses performed and statistical procedures

**Fig. 2:**
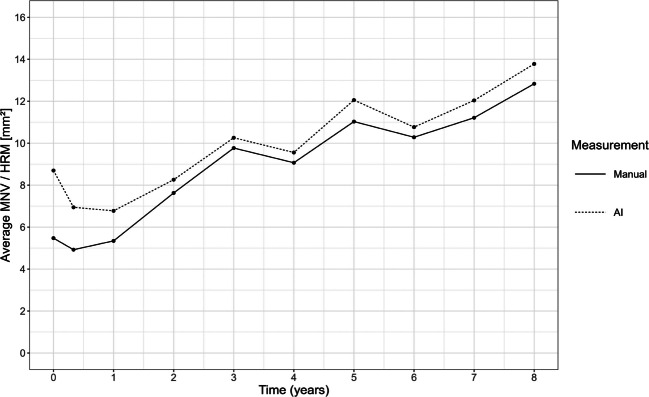
Line chart of arithmetic mean of HRM measured by AI and MNV measured manually at each time point

**Fig. 3:**
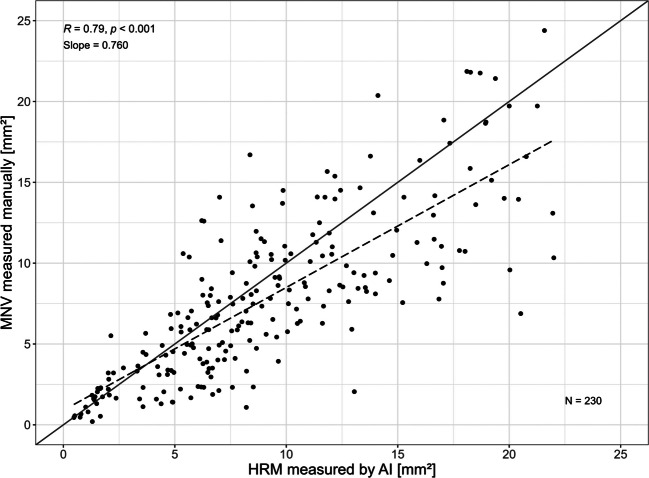
Correlation between HRM measured by AI and MNV measured manually, all time points pooled. R = Pearson correlation, including p-value testing null hypothesis: Correlation is different from zero. Slope = Estimated slope of regression line calculated by Ordinary Least Squares regression. *N* = Number of measurement pairs

**Fig. 4:**
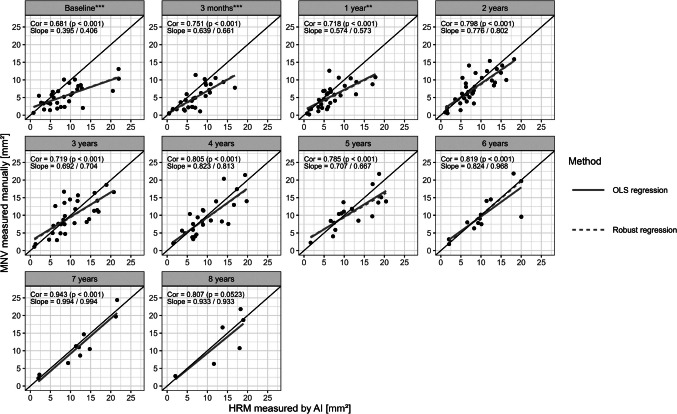
Correlation between HRM measured by AI and MNV measured manually, by time point. Annotations at each time point show Pearson correlations (Cor) including p-values testing the null hypothesis: Correlation is different from zero, and regression slopes from Ordinary Least Squares (OLS) estimation and M-Estimation (Robust). Asterisks at time points indicate p-values for an exact paired Wilcoxon two-sample test, testing the null hypothesis: Median of differences between measurements equal to zero (* = *p* < 0.05, ** = *p* < 0.01, *** = *p* < 0.001)

HRM/MNV lesion area was calculated at each time point from both manual and AI-derived segmentation masks. To obtain the 2D surface areas of HRM in mm^2^, the 3D scans were digitally projected onto the *en-face* plane (see Fig. 1). Mean HRM lesion area over time by measurement modality was visualized in a line chart (Fig. 2). A scatterplot of both manual- and AI-based measurements was generated based on pooled data across all time points (Fig. 3). Scatterplots of manually measured and AI-predicted HRM lesion areas were created for each time point separately to assess agreement over time (Fig. 4).

Pearson correlation coefficients (PCC) and ordinary least squares (OLS) regression models were computed for pooled data as well as separately for each time-point to assess the relationship between both measurement modalities. In addition, robust regression using M-estimation was applied at each time point to assess the influence of potential outliers.

## Results

Forty-two eyes from 36 patients (21 f, 15 m; mean age baseline 76.6 y) with exudative nAMD and under effective anti-VEGF treatment were analyzed. Demographic, clinical and morphological characteristics were summarized in Table 1.

**Table 1:** Demographic, clinical and morphological characteristics

Characteristic	
Number of eyes	42
Number of patients	36
Baseline age (yrs), mean ± SD	76.6 ± 6.9
Sex	
Male, no. (%)	15 (41.7%)
Female, no. (%)	21 (58.3%)
Number of injections	
Overall, mean ± SD	36.7 ± 14,2
per year, mean ± SD	7.1 ± 2.5
Follow-up time (yrs), mean ± SD (range, median)	5.3 ± 1.7 (3–8, 5.0)
Manual MNV size at baseline (mm^2^), mean ± SD	5.1 ± 3.1
AI-based HRM size at baseline (mm^2^), mean ± SD	8.6 ± 5.2
Manual MNV size at final visit (mm^2^), mean ± SD	10.5 ± 5.5
AI-based HRM size at final visit (mm^2^), mean ± SD	10.6 ± 5.5

Comparison of MNV size measurements using manual and AI-based annotations across all time points demonstrated a similar growth trend of HRM/MNV lesions over time (Fig. 2). AI-based measurements were generally slightly greater than manual annotations at most visits, with the greatest differences being observed at baseline and during the first year of treatment (Fig. 2). Despite these differences especially at the beginning of treatment, a close alignment of the size measurements over time between the two methods could be observed.

**Fig. 5:**
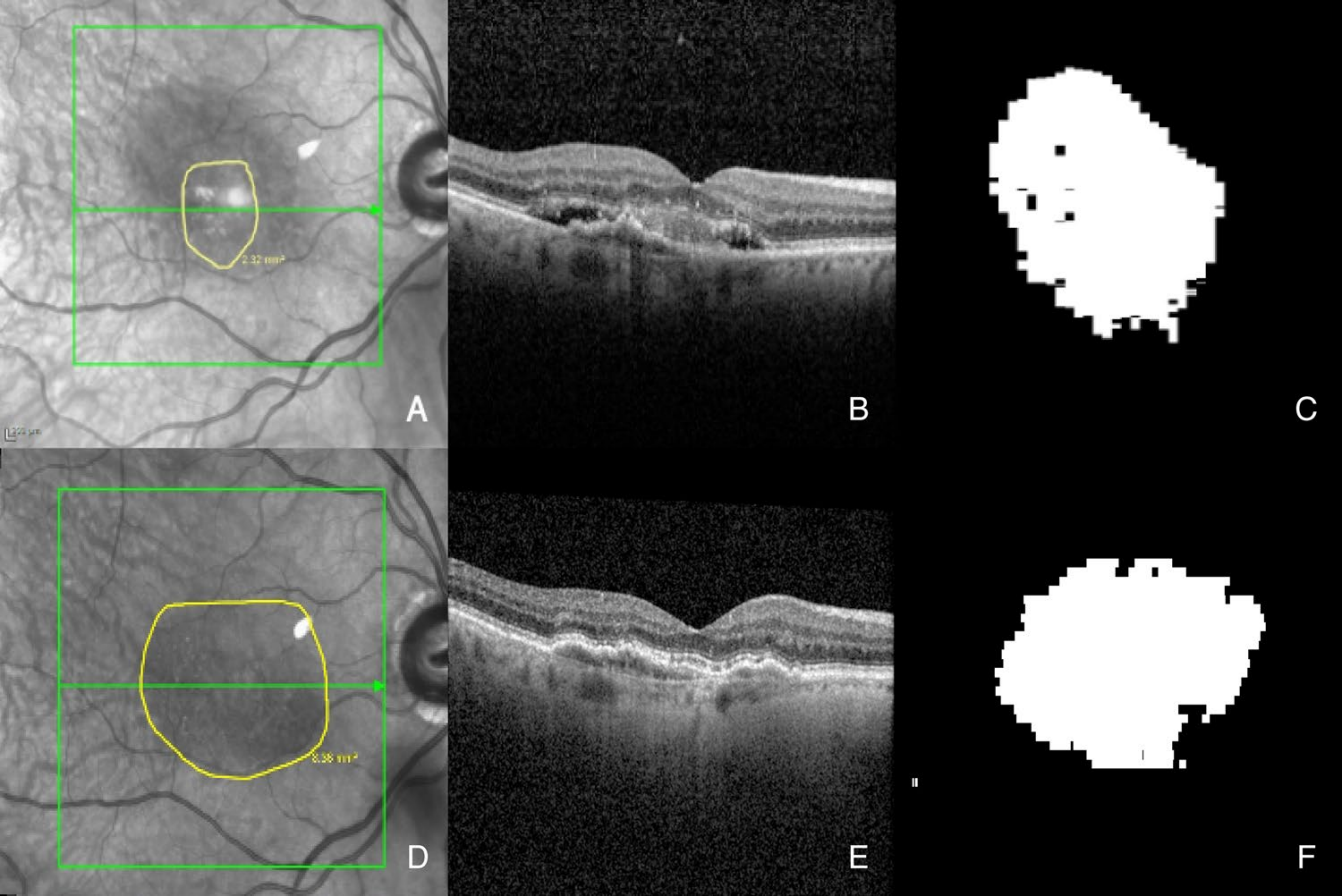
MNV/HRM with smaller manual demarcated MNV (A = baseline 2,32 mm^2^; D = final visit 8.36 mm^2^) compared to AI-based HRM at baseline (B, C = 7.58 mm^2^) and final visit (E, F = 7.13 mm^2^) due to baseline hyperreflective HRM-fluid

**Fig. 6:**
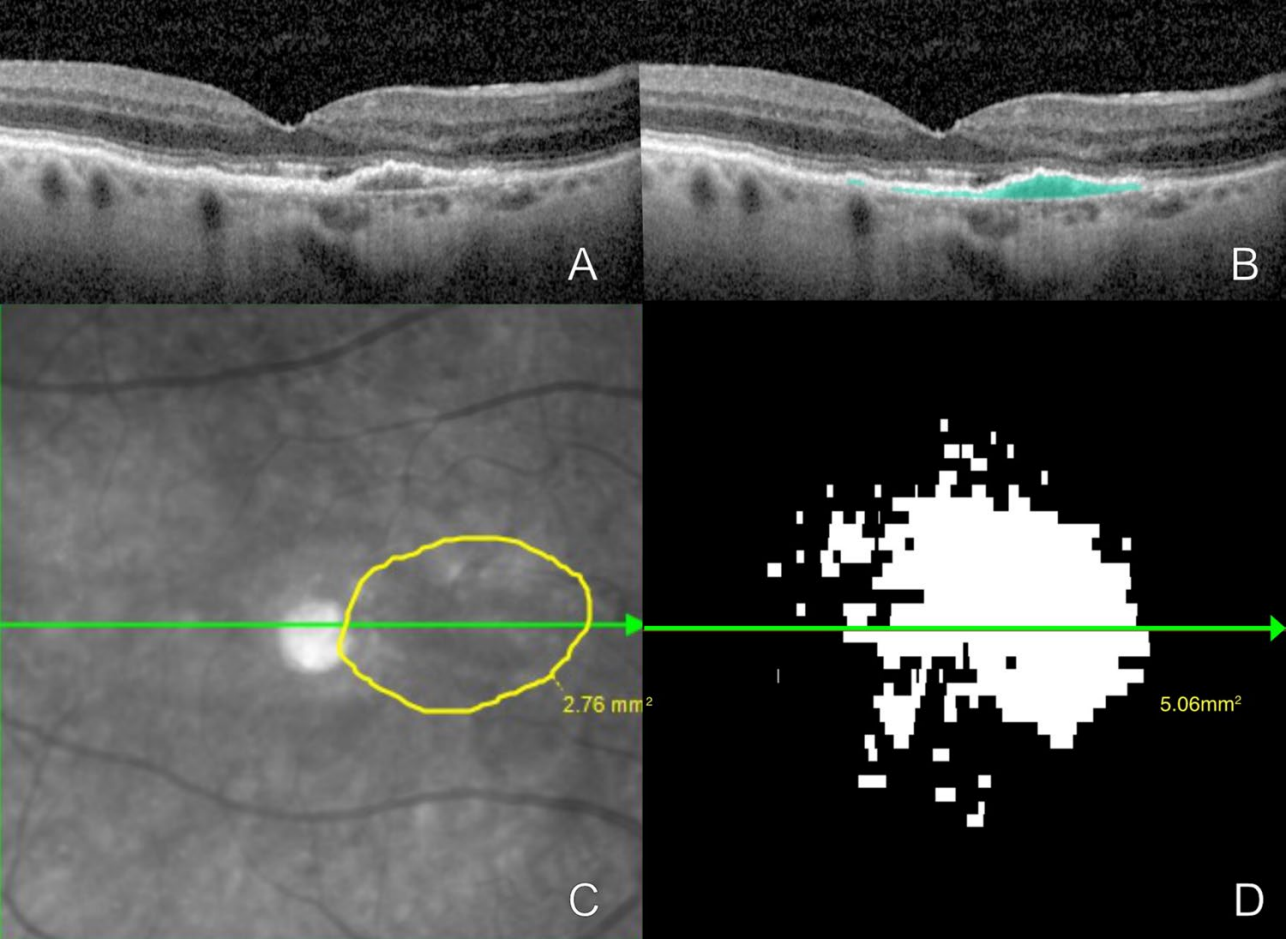
Disagreement of MNV/HRM measurement due to smaller manual MNV measurement (A = 2.76 mm^2^) compared with larger HRM size (B = 5.06 mm^2^) due to thin type 1 MNV

The difference in size measurements at baseline appears to be driven by a greater amount of hyperreflective material present at that time (Fig. 5), which was subsequently resorbed following the initial anti-VEGF therapy phase, as previously reported. In addition, flatter HRM lesions were more reliably detected by the AI algorithm than by manual annotation, contributing to the slightly larger MNV areas identified by the AI-based approach especially during follow-up (Fig. 6). Point-by-point analysis also supports this, demonstrating an significant overall correlation between AI-predicted and manually annotated HRM lesion areas across all time points (r = 0.78, *p* < 0.001) (Fig. 3).

Comparing the relationship between AI-derived and manual HRM measurements after 3 months and subsequent annual time points, strong and statistically significant correlations (p < 0.001) were observed at all time points, with coefficients ranging from R = 0.681 to R = 0.943 (Fig. 4). Accordingly, regression slopes varied: as shown in Fig. 5, the larger AI-based HRM measurements at baseline and during the first year of treatment (time points 1–3) contributed most to the discrepancies observed in the overall model. By contrast, the later time points, particularly time points 9 and 10, showed near-ideal agreement with slopes approaching 1 and minimal influence from outliers, as confirmed by ordinary least squares and robust regression estimates. This indicates that over time the manual and AI-based measurements converged.

## Discussion

The comparison between manual and AI-based analysis of *en-face* MNV/HRM revealed a high degree of similarity and concordance in measuring the size and growth of MNV lesions. In addition, point-by-point analysis showed a correlation coefficient of r = 0.78 between AI-predicted and manually measured HRM lesion areas, also indicating good agreement. A third aspect supporting the good comparability between both methods was the observation that over longer follow-up periods the larger AI-based measurements gradually converged with manual assessments. After 6 to 8 years, both methods produced highly comparable results with slope values between 0.8 and 0.9. This consistency highlights the reliability of AI-based HRM analysis for long-term monitoring of lesion size.

However, manual MNV as well as AI-based HRM measurement showed certain limitations over time. The basic assumption of this work is that HRM on OCT detected by the AI algorithm represents the sub-RPE and subretinal part of the MNV in treated nAMD. This assumption had to be made, since the AI can only detect predefined anatomical endpoints identifiable on OCT imaging and HRM is the most closely aligned feature of MNV on OCT. But especially in early phases of nAMD or in treatment-naïve cases, HRM may also represent exudation and hyperreflective fluid from the MNV. The initial variance between the AI and manual measurements can be attributed to the AI's segmentation of all hyperreflective material, which often includes a significant fluid component alongside the MNV in treatment-naive eyes. The subsequent resorption of this fluid under anti-VEGF therapy clarifies the true lesion boundaries. This dynamic offers an explanation not only for the initial discrepancy but also the eventual convergence and strong correlation between the two methods during long-term follow-up.

In order to quantify the influence of HRM corresponding to hyperreflective fluid at baseline previous studies investigated the different clinical presentation of HRM [12, 27]. In these studies, different patterns of HRM on OCT were observed: pattern 1 = no HRM, pattern 2 = subretinal HRM resolved during follow-up, pattern 3 = persistent subretinal HRM with new HRM-boundary remodeling, pattern 4 = persistent subretinal HRM without HRM-boundary remodeling. Pattern 2, which was observed in 23.1% of eyes at baseline [27], therefore described larger HRM at baseline due to hyperreflective fluid, which was resorbed after the loading phase. This effect attributes to the larger AI-based HRM lesion measurements at baseline.

Manual measurements of MNV size is also subject to inherent limitations. Although efforts were made to accurately transfer the MNV boundaries on OCT B-scans to the en-face image, this manual measurement is limited by the subjectivity of the MNV demarcation in the B-scan with subsequent delineation using the region finder software. This aspect of MNV size underestimation may be particularly important in cases of thin type 1 MNV. In these cases, the outer boundaries of the lesion were difficult to delineate, leading to greater variability. AI, on the other hand, was able to detect these boundaries more accurately and reproducibly, resulting in larger HRM lesions during follow-up compared to manual MNV measurements.

In addition, several other factors may also contribute to the discrepancies observed between manual and AI-based measurements. These include potential imbalances in the AI training dataset, variations in image quality, and challenges in defining lesion boundaries in borderline or ambiguous cases. Methodological differences may also play a role: for example, manual annotations were made on OCT volume scans and then transferred to NIR *en-face* images, while the AI made direct predictions from OCT data. Such discrepancies may introduce systematic variance.

To further improve the performance of AI-based analysis, enhancements such as expanding the training dataset to include more large lesions seem warranted.

Despite the identified limitations of both methods, the AI system demonstrated reliable longitudinal tracking of lesion progression. This indicates strong potential for automated, long-term disease monitoring in neovascular AMD.

## Conclusion

The comparison between manual MNV measurements and AI-based HRM measurements (HRM as correlate for MNV) shows substantial agreement, with AI-based measurements tending to yield slightly larger lesion areas than manual assessments in certain cases. This is likely due to the AI's improved ability to detect flat HRM regions that are thinner and more challenging to delineate through visual inspection and manual segmentation. Furthermore, in cases with substantial hyperreflective fluid at baseline, an AI-based analysis may overestimate MNV/HRM areas due to the inclusion of fluid components as parts of the MNV/HRM. Nonetheless, both methods are comparable for measuring HRM/MNV lesion size. This supports the use of AI-based algorithms for automated MNV size analysis, particularly in large patient cohorts.
